# Buffer and salt dependent reassembly pathways of *Myxococcus xanthus* encapsulin

**DOI:** 10.1002/pro.70756

**Published:** 2026-08-12

**Authors:** Varnika Yadav, Tobias Beck

**Affiliations:** ^1^ Department of Chemistry, Institute of Physical Chemistry University of Hamburg Hamburg Germany; ^2^ The Hamburg Centre for Ultrafast Imaging Hamburg Germany

**Keywords:** encapsulin, icosahedral symmetry, ionic strength, nanocompartments, protein cage assembly, self‐assembly, triangulation number

## Abstract

Protein cages that adopt multiple assembly states provide tractable systems for probing the mechanisms that govern icosahedral shell formation. The *Myxococcus xanthus* encapsulin shell protein assembles into either a *T* = 1 shell (60 subunits) or a *T* = 3 shell (180 subunits), making it an ideal model to investigate how solution conditions bias competing assembly pathways. Here, we examined the reassembly of *M. xanthus* encapsulin following complete urea‐mediated disassembly, systematically varying buffer identity (4‐(2‐hydroxyethyl)‐1‐piperazineethanesulfonic acid [HEPES], Tris, or phosphate) and ionic strength (0–1M NaCl) at pH 7.5. Assembly products were analyzed by dynamic light scattering and size‐exclusion chromatography, with selected fractions characterized by transmission electron microscopy. Buffer identity and salt concentration reproducibly determined the *T* = 1 versus *T* = 3 distribution: phosphate buffer strongly favored *T* = 1 under all conditions, Tris at low ionic strength promoted *T* = 3 formation, and increasing NaCl progressively shifted outcomes toward *T* = 1 or unassembled subunits depending on the buffer. Assembled populations remained fixed upon shell closure and did not interconvert upon buffer exchange, demonstrating kinetic trapping. Interface analysis of both assembly states reveals that *T* = 3 formation requires reversible sampling of multiple weak contacts following formation of one dominant interface, whereas *T* = 1 relies on repeated formation of a single hydrophobic interface. Together, these results show that *M. xanthus* encapsulin assembly proceeds through competing kinetic pathways, with the outcome determined by how solution conditions modulate the reversibility and selectivity of early subunit encounters.

## 1 INTRODUCTION

A defining feature of many protein cages is the existence of multiple possible assembly states. Shell architecture is commonly described by the triangulation number (*T*), which defines both subunit number and curvature, with *T* = 1 and *T* = 3 assemblies being particularly prevalent among viral capsids and related protein cages (Caspar & Klug, 1962; Johnson et al., 2005). While high‐resolution structural studies have provided detailed descriptions of these end states, much less is known about how solution conditions bias assembly pathways toward one geometry over another (Zlotnick, 1994). In particular, it remains unclear whether distinct shell architectures reflect thermodynamic equilibria between assembled states or are instead determined by kinetic decisions made during early stages of nucleation and growth (Hagan & Chandler, 2006; Perlmutter & Hagan, 2015; Zlotnick, 1994).

Encapsulins provide a useful model system for addressing these questions. Encapsulins are protein nanocompartments found widely in bacteria and archaea that assemble into icosahedral shells and selectively encapsulate cargo proteins via short cargo‐loading peptides, with growing interest as platforms for biotechnology and nanomedicine applications (Giessen, 2022; Giessen & Silver, 2017; Jones & Giessen, 2021; Nichols et al., 2017). Their shell proteins adopt the HK97‐like capsid fold, linking encapsulins structurally and evolutionarily to viral capsids while supporting diverse metabolic functions (Giessen, 2022; Wikoff et al., 2000). Encapsulin shells contain pores located at symmetry axes that permit molecular exchange, allowing encapsulated enzymes to remain active while sequestered from the cytosol (Nichols et al., 2017; Sutter et al., 2017). Encapsulins are also robust assemblies that can undergo controlled disassembly and reassembly, enabling direct experimental interrogation of assembly pathways (Künzle et al., 2018; Moon et al., 2014).

Among encapsulins, the system from *Myxococcus xanthus* is notable for its structural plasticity. A single shell protein assembles into two distinct architectures: a *T* = 1 shell composed of 60 subunits and a *T* = 3 shell composed of 180 subunits (Eren et al., 2022). In vivo, the *T* = 3 state predominates and encapsulates multiple ferritin‐like proteins involved in iron storage and oxidative stress resistance, whereas in recombinant systems lacking cargo, both geometries can form (Eren et al., 2022; Giessen et al., 2019). Structural studies have shown that this polymorphism arises from conformational flexibility in the E‐loop region of the shell protein, enabling the same protomer to support different curvatures and interface arrangements (Eren et al., 2022). Engineering studies have further demonstrated that encapsulin shell properties, including pore characteristics and assembly behavior, can be modulated through targeted protein modifications (Kwon et al., 2024). These observations suggest that *M. xanthus* encapsulin assembly may proceed through competing pathways rather than through equilibration between closed shells (Hagan & Chandler, 2006; Perlmutter & Hagan, 2015).

Here, we examine how buffer identity and ionic strength bias the reassembly of *M. xanthus* encapsulin following complete disassembly in 8M urea. Reassembly was initiated at pH 7.5 in HEPES, Tris, and phosphate buffers across a broad range of NaCl concentrations, and assembly products were analyzed using dynamic light scattering and size‐exclusion chromatography (SEC). For selected conditions, transmission electron microscopy (TEM) was used to confirm *T* = 1 and *T* = 3 assemblies. By combining reassembly experiments with structural interface analysis, we show that assembly outcomes are determined during early nucleation, remain kinetically trapped once shells close, and can be rationalized by the distinct interface hierarchies underlying the *T* = 1 and *T* = 3 architectures.

## 2 METHODS

### 2.1 Protein expression and purification

The *M. xanthus* encapsulin shell protein was expressed in *Escherichia coli* BL21(DE3) cells transformed with the corresponding expression plasmid. Cells were grown in terrific broth supplemented with ampicillin (150 μg/mL) at 37°C to an OD_600_ of ~0.6, and protein expression was induced with isopropyl β‐D‐1‐thiogalactopyranoside (IPTG; final concentration 0.2 mM). Expression was carried out at 18°C for approximately 60 h. Cells were harvested by centrifugation and stored at −20°C until use. Cell pellets were thawed and resuspended in 25 mL of lysis buffer (20 mM Tris, 200 mM NaCl, pH 8.0) supplemented with lysozyme (0.6 mg/mL), DNase I (0.2 mg/mL), and RNase A (0.4 mg/mL). Resuspended pellets were lysed by sonication on ice at 60% amplitude, in 8–12 cycles of 59 s pulse and 59 s pause per pellet. Lysates were clarified by centrifugation at 14,000 × g for 15 min at 4°C. To facilitate further removal of nucleic acids, MgCl_2_ and CaCl_2_ were added to the clarified lysate to final concentrations of 2.5 and 0.5 mM, respectively, and samples were incubated at 37°C for 2 h, followed by centrifugation (14,000 × g, 15 min, 4°C) to remove precipitated material. Remaining soluble contaminants were removed by heat precipitation at 60°C for 15 min in a water bath, after which the solution was cooled on ice and clarified by centrifugation (14,000 × g, 15 min, 4°C). Proteins were precipitated by addition of solid ammonium sulfate to 70% saturation at 4°C with gentle stirring, and the precipitate was collected by centrifugation (14,000 × g, 20 min, 4°C).

The protein pellet was resuspended in 20 mM Tris buffer (pH 8.0) and purified by anion‐exchange chromatography using a HiTrap Q HP column (Cytiva) with a linear NaCl gradient from 0 to 1M. Fractions containing *M. xanthus* encapsulin were concentrated and further purified by SEC on columns equilibrated in 20 mM Tris, 200 mM NaCl, pH 8.0. A HiPrep Sephacryl S‐500 HR column (Cytiva) was used for large‐scale preparations and a Superose 6 Increase column (Cytiva) for smaller sample volumes.

### 2.2 Disassembly and reassembly of encapsulin cages

Encapsulin cages (predominantly *T* = 3) were precipitated by addition of polyethylene glycol (PEG) 8000 and NaCl to final concentrations of 10% (w/v) and 0.5M, respectively. Samples were incubated on ice for 1 h and centrifuged at 21,000 × g for 45 min. The supernatant was removed, and the pellet was gently resuspended in freshly prepared 8M urea dissolved in 20 mM phosphate buffer containing 0.1 M NaCl (pH 7.5), yielding a final protein concentration of approximately 20 μM monomer equivalent. Samples were incubated on ice for 3 h to allow complete disassembly, which was confirmed by dynamic light scattering (DLS).

Reassembly was initiated by dilution of the denatured protein into reassembly buffers (20 mM HEPES, Tris, or phosphate, pH 7.5) containing defined NaCl concentrations (0, 0.1, 0.5, or 1.0M). Urea removal, buffer exchange, and concentration back to a final volume of approximately 1 mL were performed simultaneously by centrifugal filtration at 4°C using 10 kDa molecular weight cutoff filters, during which reassembly proceeded. Formation of higher‐order assemblies was confirmed by DLS. Samples were stored at 4°C overnight to allow equilibration prior to SEC analysis the following day.

### 2.3 Dynamic light scattering

DLS measurements were performed to assess the hydrodynamic size distributions of encapsulin assemblies before disassembly, after disassembly, and following reassembly. Measurements were carried out at 25°C using a Zetasizer Nano S (Malvern Instruments) equipped with a 633 nm He–Ne laser, with samples equilibrated for at least 120 s prior to data acquisition. *Z*‐average hydrodynamic diameters and polydispersity indices were obtained from cumulant analysis of the intensity‐weighted correlation data, while volume‐weighted distributions were used to visualize the dominant size populations.

### 2.4 Size‐exclusion chromatography

SEC was used as the primary method to resolve distinct encapsulin assembly states following reassembly. SEC was performed at room temperature using a Superose 6 Increase column (Cytiva) on an ÄKTA FPLC system equilibrated in the corresponding reassembly buffer, at a flow rate of 0.5 mL/min. All buffers were adjusted to pH 7.5 at room temperature prior to use. Samples were clarified by centrifugation prior to injection, and elution profiles were monitored by absorbance at 280 nm.

### 2.5 Transmission electron microscopy

TEM was used to directly visualize encapsulin assemblies and validate SEC peak assignments. Fractions corresponding to the ~9 and ~13 mL SEC peaks were collected and analyzed by negative‐stain TEM. Carbon‐coated copper grids (400 mesh; Ted Pella) were used for imaging. Samples were applied to grids and either air‐dried (unstained) or negatively stained using 2% (w/v) uranyl acetate. For stained samples, grids were incubated on sample droplets for 1 min, washed with ultrapure water, stained for 60 s, blotted, and air‐dried. Imaging was performed using a JEOL JEM‐1011 transmission electron microscope operated at 100 kV.

### 2.6 Structural interface analysis

Interface analysis was performed using the Protein Data Bank (PDB) ePISA (Proteins, Interfaces, Structures and Assemblies) server (https://www.ebi.ac.uk/pdbe/pisa/) on the deposited coordinates of the *T* = 3 (PDB: 4PT2) and *T* = 1 (PDB: 7S21) *M. xanthus* encapsulin assemblies (Krissinel & Henrick, 2007). For the *T* = 3 assembly, the three unique interfaces of the asymmetric unit (A–B, A–P, and B–P) were analyzed. For the *T* = 1 assembly, the four representative interfaces with the largest buried surface areas were tabulated. Reported buried surface areas, solvation free energy gains, hydrogen bonds, and salt bridges are taken directly from the proteins, interfaces, strcutures and assemblies (PISA) output.

## 3 RESULTS AND DISCUSSION

**FIGURE 1:**
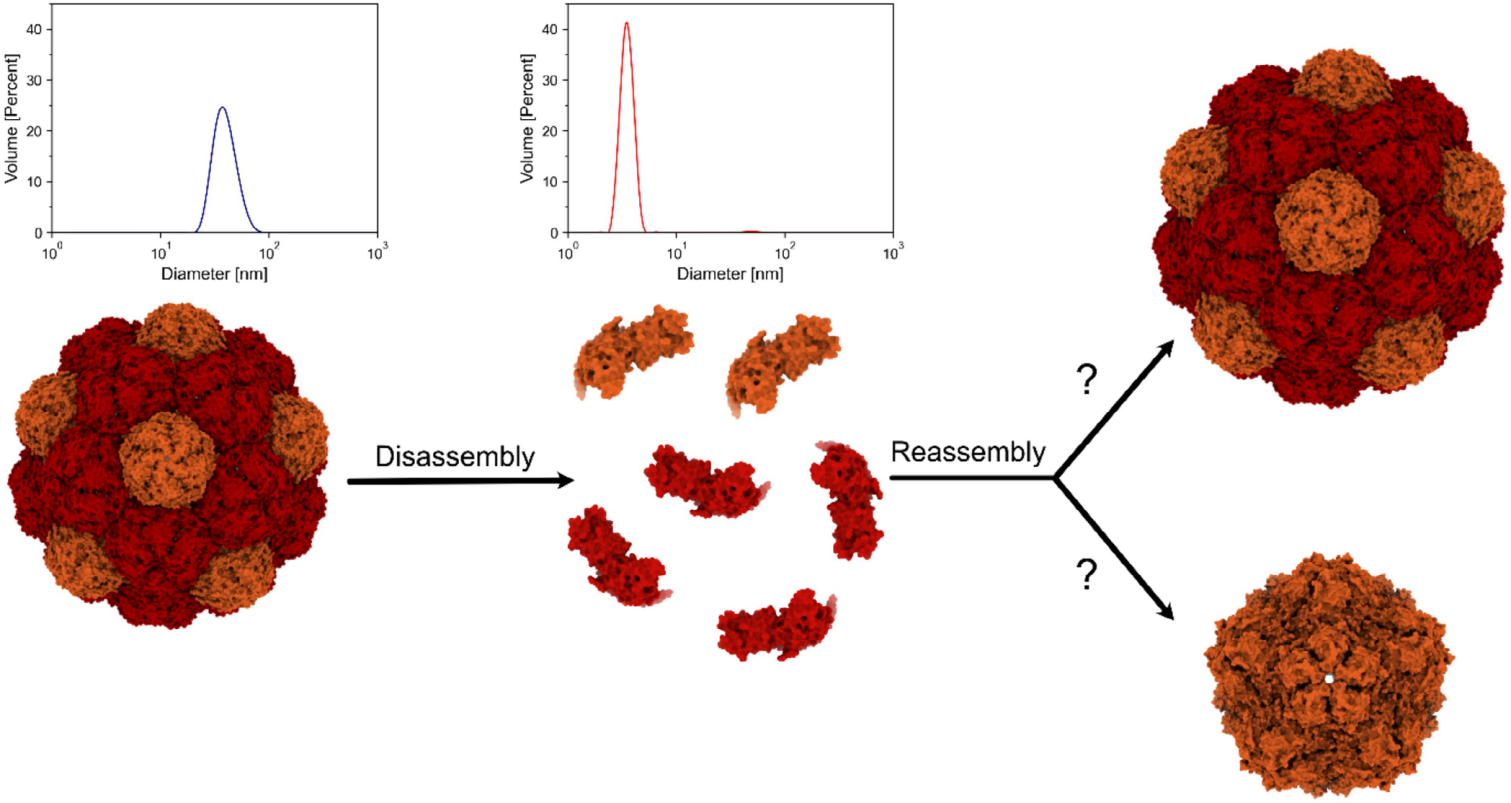
Disassembly of *Myxococcus xanthus* encapsulin and reassembly into alternative shell architectures. Dynamic light scattering volume‐weighted size distributions confirm the native cage population (blue trace, upper left) and efficient disassembly into monomeric or low‐order oligomeric subunits (red trace, middle). Disassembled subunits can subsequently reassemble into either *T* = 3 (180 subunits, upper right) or *T* = 1 (60 subunits, lower right) shells depending on solution conditions. In the structural models, hexameric capsomers are shown in maroon and pentameric capsomers in orange. Structures shown correspond to PDB entries 4PT2 (*T* = 3) and 7S21 (*T* = 1).

A schematic illustration of encapsulin disassembly and reassembly into alternative shell architectures is shown in Figure 1. Encapsulin cages (*T* = 3) were disassembled by PEG precipitation followed by incubation in 8M urea for 3 h at 4°C, adapting previously reported disassembly conditions to ensure complete dissociation (Van de Steen et al., 2024). DLS confirmed efficient disassembly, yielding a dominant population at small hydrodynamic diameters consistent with monomeric or low‐order oligomeric subunits (Figure 1).

Reassembly was performed in buffers of identical pH but differing chemical composition (HEPES, Tris, and phosphate), with NaCl concentrations varied from 0 to 1M. DLS measurements after reassembly confirmed the formation of large assemblies in solution under all conditions tested (Figure S1). *Z*‐average hydrodynamic diameters and polydispersity indices for all conditions are summarized in Table S1. Note that the *Z*‐average values in Table S1 are derived from the intensity‐weighted distribution, whereas the distributions shown in Figure S1 are volume‐weighted; intensity weighting emphasizes larger species, while volume weighting gives a more representative view of smaller assemblies, so the two should be compared with this difference in mind.

However, because DLS cannot resolve coexisting encapsulin assemblies with similar hydrodynamic diameters, SEC was used as the primary method to distinguish between *T* = 1, *T* = 3, and unassembled species. Assembly products were analyzed by SEC using a Superose 6 Increase column. Under all conditions tested, three reproducible species were observed: A large particle eluting at approximately 9 mL corresponding to *T* = 3 cages, a smaller particle eluting at approximately 13 mL corresponding to *T* = 1 cages, and a late‐eluting peak corresponding to unassembled subunits or small oligomers. The different *T* states (*T* = 1 and *T* = 3 assemblies) were assigned based on elution values compared with established literature values and were independently validated by TEM during purification of *M. xanthus* encapsulin, where these populations reproducibly elute at the same volumes (Figure S2).

**FIGURE 2:**
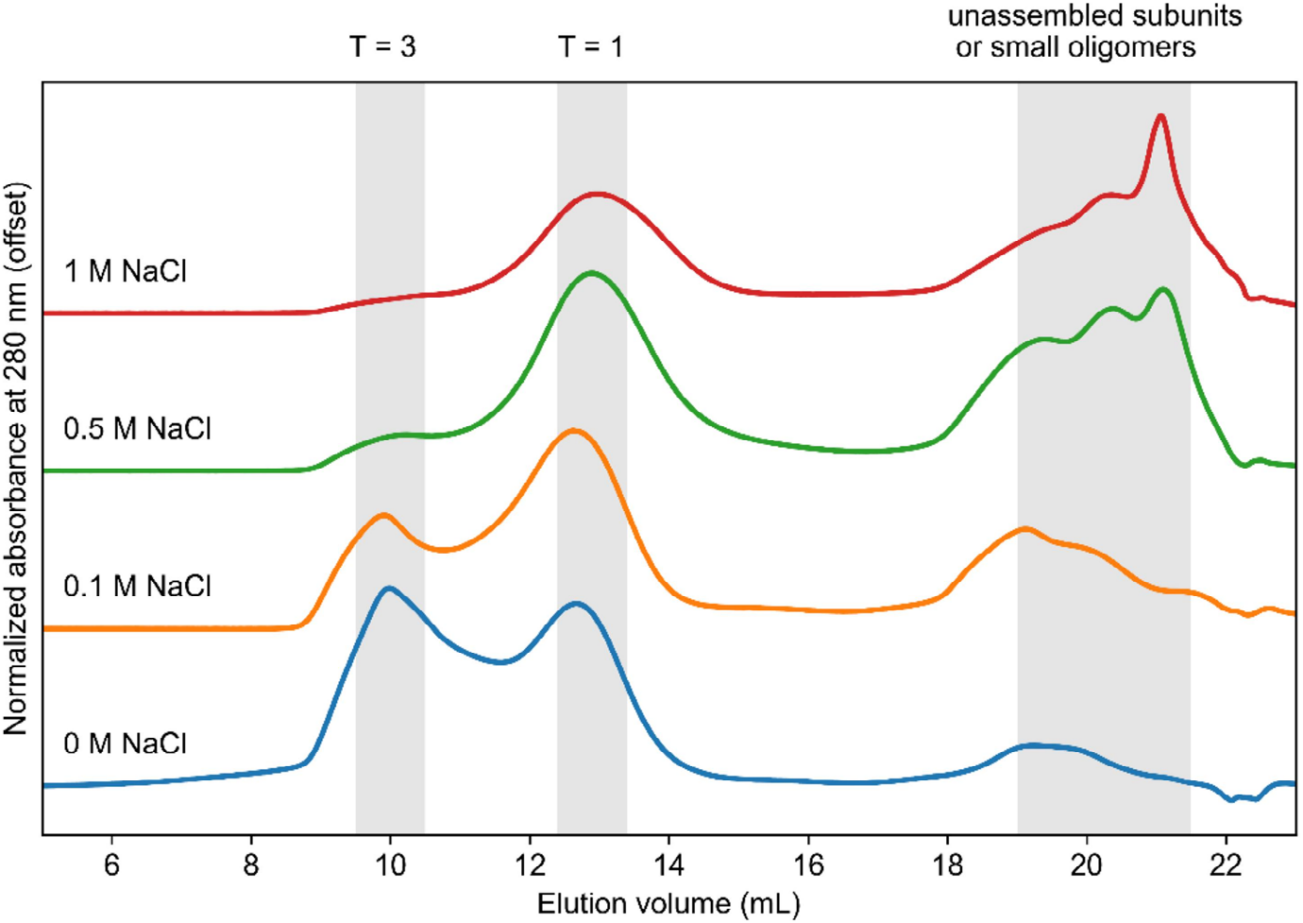
Normalized size‐exclusion chromatograms of *Myxococcus xanthus* encapsulin following reassembly in HEPES buffer with four different NaCl concentrations: 0M (blue), 0.1M (orange), 0.5M (green), and 1M (red). Peaks eluting at approximately 9 mL, 13 mL, and late retention correspond to *T* = 3 cages, *T* = 1 cages, and unassembled subunits, respectively. Absorbance was monitored at 280 nm.

In HEPES buffer, reassembly in the absence of NaCl produced comparable amounts of *T* = 1 and *T* = 3 cages (Figure 2). Increasing NaCl concentration from 0.1 to 0.5M progressively enriched the *T* = 1 population while reducing *T* = 3 abundance. At 1M NaCl, the subunit peak dominated, with only minor amounts of *T* = 1 and little to no detectable *T* = 3 species.

**FIGURE 3:**
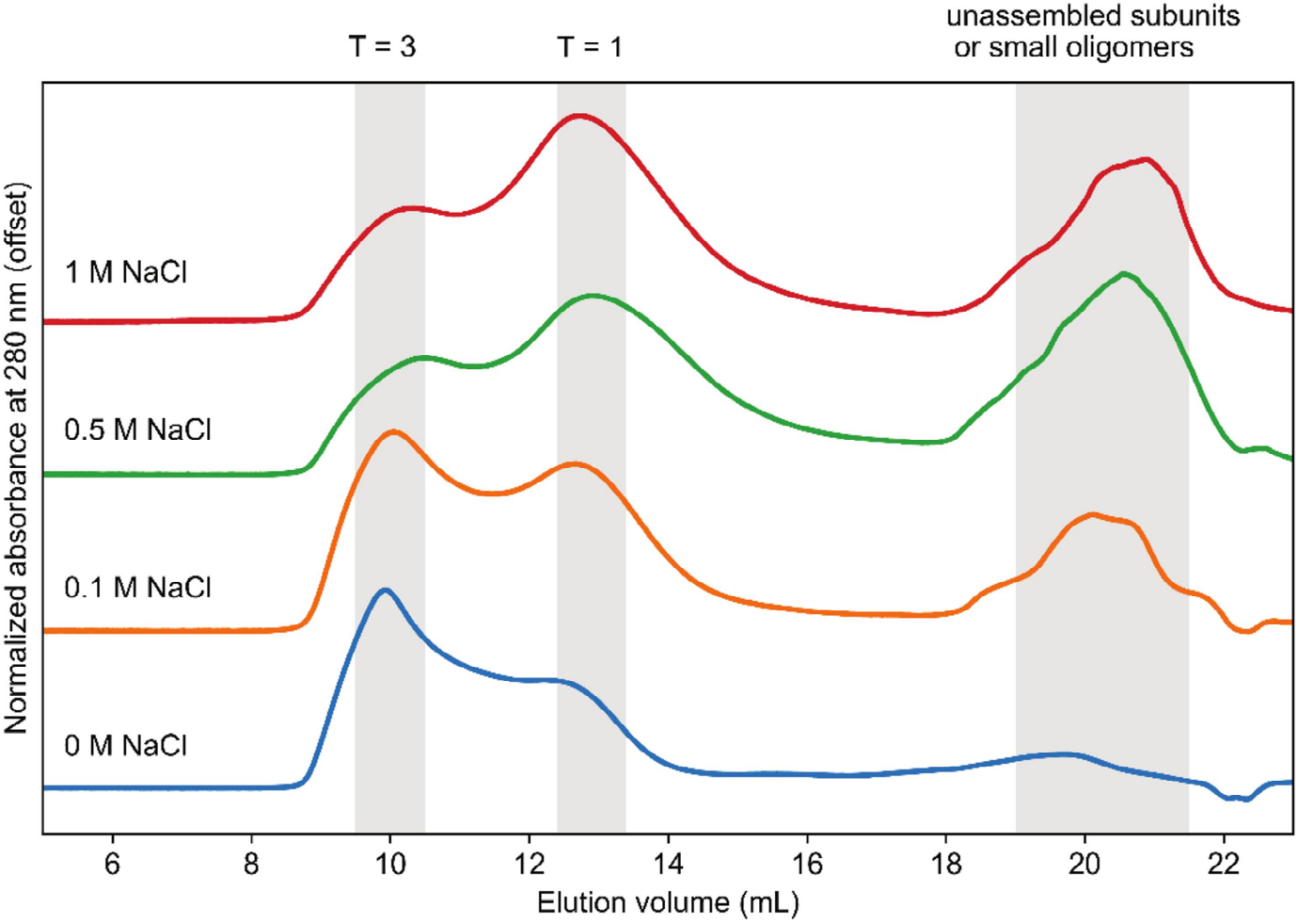
Normalized size‐exclusion chromatograms of *Myxococcus xanthus* encapsulin following reassembly in Tris buffer with four different NaCl concentrations: 0M (blue), 0.1M (orange), 0.5M (green), and 1M (red). Peaks eluting at approximately 9 mL, 13 mL, and late retention correspond to *T* = 3 cages, *T* = 1 cages, and unassembled subunits, respectively. Absorbance was monitored at 280 nm.

In Tris buffer, reassembly without added NaCl strongly favored formation of *T* = 3 cages, with minimal accumulation of subunits (Figure 3). Addition of 0.1M NaCl shifted the distribution toward *T* = 1 dominance. At 0.5M NaCl, unassembled subunits became the major species and both cage populations were substantially reduced. At 1M NaCl, *T* = 1 cages again predominated, although a significant fraction of subunit material remained.

**FIGURE 4:**
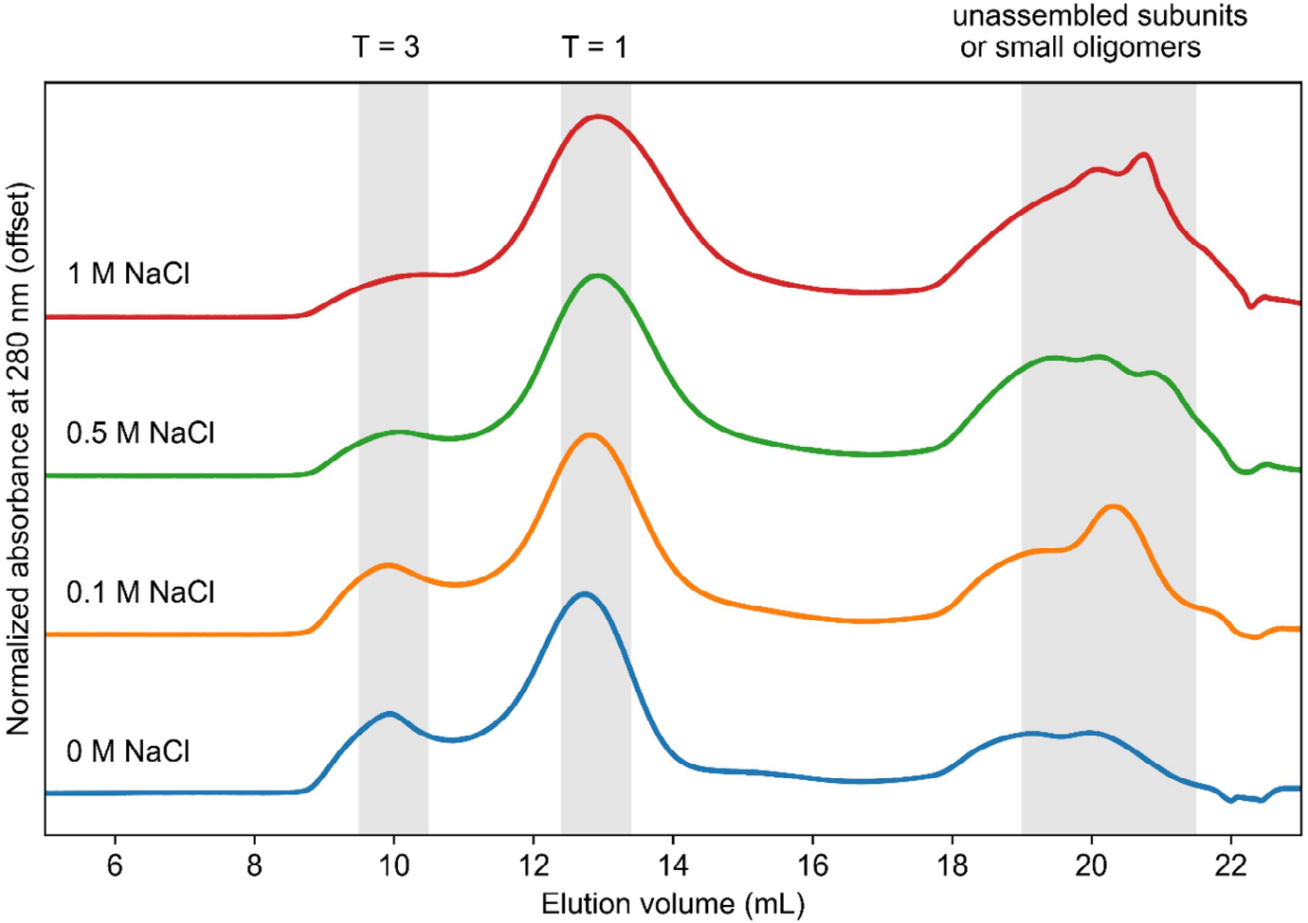
Normalized size‐exclusion chromatograms of *Myxococcus xanthus* encapsulin following reassembly in phosphate buffer with four different NaCl concentrations: 0M (blue), 0.1M (orange), 0.5M (green), and 1M (red). Peaks eluting at approximately 9 mL, 13 mL, and late retention correspond to *T* = 3 cages, *T* = 1 cages, and unassembled subunits, respectively. Absorbance was monitored at 280 nm.

In phosphate buffer, *T* = 1 cages dominated under all salt conditions examined. Even in the absence of NaCl, *T* = 1 was the major assembly product, with only minor contributions from *T* = 3 cages and subunits (Figure 4). Increasing NaCl concentration to 0.1–0.5M further suppressed *T* = 3 formation. At 1M NaCl, subunit accumulation increased and overall cage yield decreased.

**FIGURE 5:**
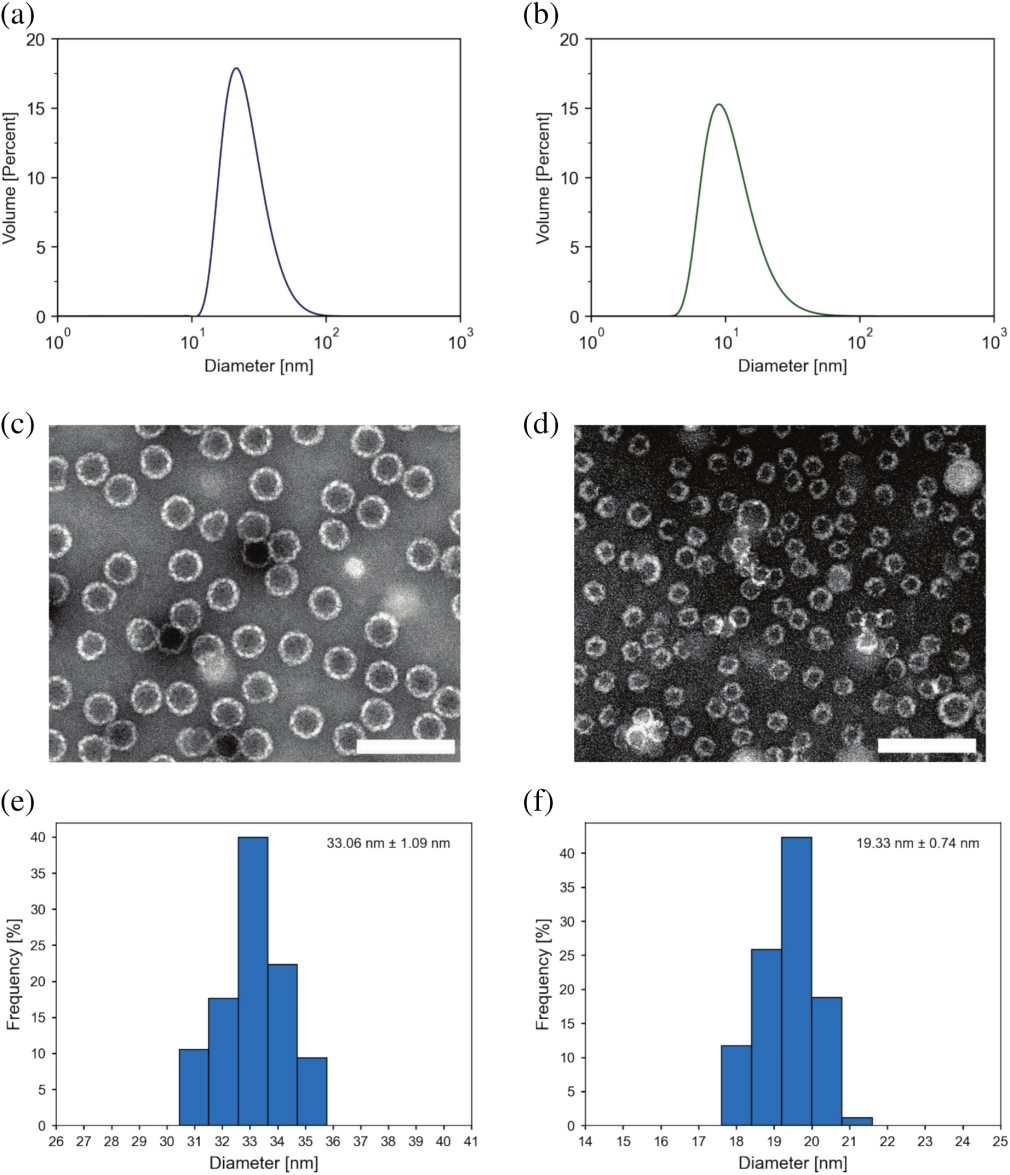
Structural validation of *T* = 3 and *T* = 1 encapsulin assemblies isolated by size‐exclusion chromatography. (a) Volume‐weighted dynamic light scattering size distribution of the *T* = 3 fraction. (b) Volume‐weighted dynamic light scattering size distribution of the *T* = 1 fraction. (c) Negative‐stain transmission electron microscopy (TEM) of the ~9 mL peak, showing predominantly intact *T* = 3 state. (d) Negative‐stain TEM of the ~13 mL peak, enriched in *T* = 1 assemblies. Scale bars, 100 nm. (e) Diameter distribution measured from negative‐stain TEM for the *T* = 3 fraction (mean 33.06 ± 1.09 nm, *n* = 100). (f) Diameter distribution measured from negative‐stain TEM for the *T* = 1 fraction (mean 19.33 ± 0.74 nm, *n* = 100).

Negative‐stain TEM of post‐SEC fractions from the 0M NaCl HEPES condition revealed uniform populations of intact cages for both the *T* = 3 and *T* = 1 fractions (Figure 5a,b). Volume‐weighted DLS of the same fractions gave single, well‐resolved size distributions consistent with assembled cages (Figure 5c,d). Particle diameters measured from the TEM images using ImageJ were 33.06 ± 1.09 nm for the *T* = 3 fraction and 19.33 ± 0.74 nm for the *T* = 1 fraction (Figure 5e,f), in good agreement with the expected dimensions of the two shells. Together these measurements serve as structural benchmarks for the SEC peak assignments. TEM was not repeated across all buffer and salt conditions because SEC elution volumes were reproducible and consistent with these validated assignments.

A consistent trend emerges across buffer systems: increasing NaCl concentration preferentially reduces the *T* = 3 population while having a comparatively smaller effect on the *T* = 1 population, with a corresponding increase in unassembled subunits at the highest ionic strength tested. Quantification of integrated SEC peaks, expressed as a fraction of the combined assembled signal, confirms this pattern across all conditions (Figure S3). Together with the buffer‐dependent differences described above, these results show that buffer identity and ionic strength systematically bias the assembly outcome toward either the *T* = 1 or *T* = 3 architecture, with the *T* = 3 pathway being the more salt‐sensitive of the two.

The experiments above show which architecture forms under a given condition, but not whether that architecture is locked in once assembled. For capsid and capsid‐like systems more generally, assembly is known to proceed under kinetic rather than thermodynamic control, with the outcome determined during early nucleation rather than by equilibration between completed shells (Endres & Zlotnick, 2002; Hagan & Chandler, 2006; Perlmutter & Hagan, 2015; Zlotnick, 1994).

To test directly whether assembled cages are kinetically trapped, encapsulin was reassembled in HEPES, Tris, or phosphate and then buffer‐exchanged into an alternative buffer, and the assembly distribution was compared by SEC before and after exchange (Figure S4). These experiments used a protein preparation stored for approximately 12 months, which on its own favored *T* = 1 more strongly across all buffers than the original material, although the buffer‐dependent trend was preserved, with Tris retaining the highest *T* = 3 content. If one assumes that completed cages interconvert in response to buffer, exchange into a *T* = 3‐favoring buffer would increase the *T* = 3 population at the expense of *T* = 1, and exchange into a *T* = 1‐favoring buffer would do the reverse. Instead, we found that both the *T* = 3 and *T* = 1 populations increased after exchange while the unassembled subunit fraction decreased, in both exchange directions (Figure S5). This pattern is inconsistent with interconversion between assembled states and instead reflects continued assembly of residual subunits during handling. Importantly, the ratio of *T* = 3 to *T* = 1 among assembled cages remained essentially constant in every condition (Table S2), confirming that completed shells retain their architecture once formed.

Structural analysis helps explain this behavior. PISA analysis shows that the *T* = 3 assembly is stabilized by one large and specific A–B interface that buries approximately 1560–1600 Å^2^ per subunit and contains hydrogen bonds and a salt bridge, while the remaining interfaces required for shell completion are much smaller (∼650 and ∼280 Å^2^) and weaker (Eren et al., 2022) (Tables S3 and S4). Formation of a *T* = 3 shell therefore requires correct formation of the dominant interface followed by successful engagement of multiple weak contacts, which depends on reversible sampling during assembly (Hagan & Chandler, 2006; Rapaport, 2008). In contrast, the *T* = 1 assembly relies on repeated formation of a single dominant interface that buries approximately 1120 Å^2^ per subunit and is stabilized mainly by hydrophobic interactions, without detectable hydrogen bonds or salt bridges (Table S5) (Eren et al., 2022). This interface can form readily and supports efficient formation of compact pentameric nuclei (Zlotnick, 1994). We note that buried surface area is a useful but coarse descriptor of interface strength and does not capture all energetic contributions; the directional polar contacts at the *T* = 3 interface (hydrogen bonds and a salt bridge) and the predominantly hydrophobic character of the *T* = 1 interface likely contribute differently to binding free energy in ways not fully reflected by surface area alone.

At the residue level, the dominant *T* = 3 A–B interface is stabilized by a salt bridge between ASP110 and LYS270 and by 14 hydrogen bonds involving residues TYR89, LYS90, GLN124, SER112, and ASN220 of one subunit contacting partner residues on the adjacent subunit, with full atom‐level details listed in Tables S6 and S7. In contrast, the dominant *T* = 1 interface lacks both hydrogen bonds and salt bridges, consistent with primarily hydrophobic stabilization. This residue‐level distinction provides a structural basis for the differential sensitivity of the two assembly pathways to electrostatic screening.

Salt concentration affects assembly by changing how subunits interact during early encounters (Endres & Zlotnick, 2002). At the ionic strengths tested in this study, the Debye screening length decreases from approximately 3 nm at 0M NaCl to approximately 0.3 nm at 1M NaCl, spanning the range over which long‐range orientational electrostatic interactions transition to fully screened, short‐range contacts. At low ionic strength, these interactions reduce the number of productive encounters but help subunits align correctly before forming stable contacts, which favors formation of the large, specific interface required for *T* = 3 assembly (Hagan & Chandler, 2006; Perlmutter & Hagan, 2015). At moderate salt, partial electrostatic screening reduces the long‐range orientational steering between approaching subunits while preserving the short‐range contacts required for productive binding. This shifts the balance from selective geometry‐dependent contacts to less specific encounters favoring rapid stabilization of the compact hydrophobic interface that defines the *T* = 1 pathway (Zlotnick, 1994). At high salt concentrations, electrostatic guidance is strongly reduced, leading to frequent but poorly oriented encounters that result in unproductive interactions and reduced overall assembly efficiency (Rapaport, 2008).

The specific assembly intermediates underlying these pathways, whether dimers, trimers, pentamers, or higher‐order capsomers, have not been definitively established for *M. xanthus* encapsulin and remain an open question. By analogy to viral capsid systems, productive nucleation typically proceeds through small oligomeric intermediates whose stability and geometry are influenced by solution conditions; however, direct identification of such intermediates for *M. xanthus* encapsulin will require approaches such as time‐resolved scattering, mass photometry, or native mass spectrometry, which are beyond the scope of the present study.

Buffer identity further influences assembly by modifying electrostatic screening and hydration effects beyond bulk ionic strength. Phosphate buffer strongly favored *T* = 1 assembly under all conditions tested, whereas Tris at low salt favored *T* = 3. Several non‐exclusive mechanisms could contribute to these differences, including ion‐specific effects on protein hydration, electrostatic screening contributions from the buffer ions themselves, and the possibility of weak specific interactions with the shell protein (Collins, 2006). Distinguishing among these contributions would require systematic comparison with other anions of varying Hofmeister character and is identified in the future directions paragraph as an extension of this work.

The dependence of assembly outcomes on solution conditions observed here is broadly consistent with previous reassembly studies of *M. xanthus* encapsulin. Boyton et al. reported reassembly experiments in low‐ionic strength HEPES buffer that produced predominantly *T* = 3 assemblies, consistent with our observations in HEPES at low NaCl (Boyton et al., 2022). Van de Steen et al. (2024) performed reassembly in Tris buffer with moderate salt concentrations and reported mixed *T* = 1 and *T* = 3 populations, consistent with our finding that Tris with added NaCl shifts the distribution toward *T* = 1 dominance. Both prior studies also report variability between preparations and the presence of intermediate‐sized species. The systematic mapping presented here provides a framework in which this variability is expected: conditions close to the boundary between competing pathways are sensitive to small changes in solution chemistry, protein concentration, and sample history (Perlmutter & Hagan, 2015). A more rigorous direct comparison would require quantitative matching of buffer concentration, pH, ionic strength, protein concentration, and temperature, which is complicated by differences in reported experimental conditions across studies and is identified in our Future Directions as a target for follow‐up work.

Several extensions of this work would refine the mechanistic interpretation presented here. Finer titration of NaCl concentration around the apparent transitions, exploration of pH dependence, and variation of reassembly temperature would allow construction of a more complete phase diagram and would help further distinguish kinetic from thermodynamic contributions to assembly. Time‐resolved measurements would complement the buffer exchange experiments presented here by probing the kinetic stability of the assembled states as they form. Comparison with other anions of varying Hofmeister character, including sulfate or chaotropes, would clarify whether the phosphate‐specific effect reflects general electrostatic and hydration contributions or involves specific ion–protein interactions. Orthogonal techniques such as mass photometry, native mass spectrometry, analytical ultracentrifugation, and small‐angle x‐ray scattering, together with replicate measurements and formal statistical analysis, would provide quantitative population estimates with associated uncertainty and characterize potential assembly intermediates. Finally, examining reassembly in the presence of native or engineered cargo proteins would test the influence of scaffolding interactions on *T*‐number selection (Jones & Giessen, 2021; Van de Steen et al., 2024).

## 4 CONCLUSIONS

The reassembly of *M. xanthus* encapsulin into *T* = 1 or *T* = 3 shells is not determined by thermodynamic equilibration between completed assemblies but is instead fixed at the nucleation stage by the solution conditions present during early subunit encounters. Buffer identity and ionic strength together control whether assembly proceeds through the reversible, orientation‐selective interactions required for *T* = 3 lattice completion or through rapid stabilization of the compact hydrophobic interface that defines the *T* = 1 pathway. Once a shell has closed, the two states are kinetically stable and do not interconvert. This behavior can be rationalized by the distinct interface hierarchies of the two architectures: *T* = 3 depends on successful engagement of multiple weak contacts after formation of one dominant interface, making it sensitive to conditions that permit reversible sampling, while *T* = 1 is built from repeated formation of a single robust interface and is therefore favored whenever electrostatic guidance is screened. These findings establish a practical framework for controlling encapsulin shell geometry through solution chemistry, with practical implications for the use of encapsulins as programmable nanocompartments in biotechnology and materials science.

## AUTHOR CONTRIBUTIONS

**Varnika Yadav**: Investigation; methodology; formal analysis; visualization; writing—original draft. **Tobias Beck**: Conceptualization; supervision; funding acquisition; writing—review and editing.

## Supporting information


**Data S1.** Supporting Information.

## Data Availability

The data that support the findings of this study are available from the corresponding author upon reasonable request.
