# Supplementary material for: Buffer and salt dependent reassembly pathways of Myxococcus xanthus encapsulin
Source: Protein Sci. 2026 Aug 12;35(9):e70756. doi: 10.1002/pro.70756 (PMC13468902; doi:10.1002/pro.70756)
Supplement: Supplementary file 1 [file PRO-35-e70756-s001.pdf]

Supplementary Information

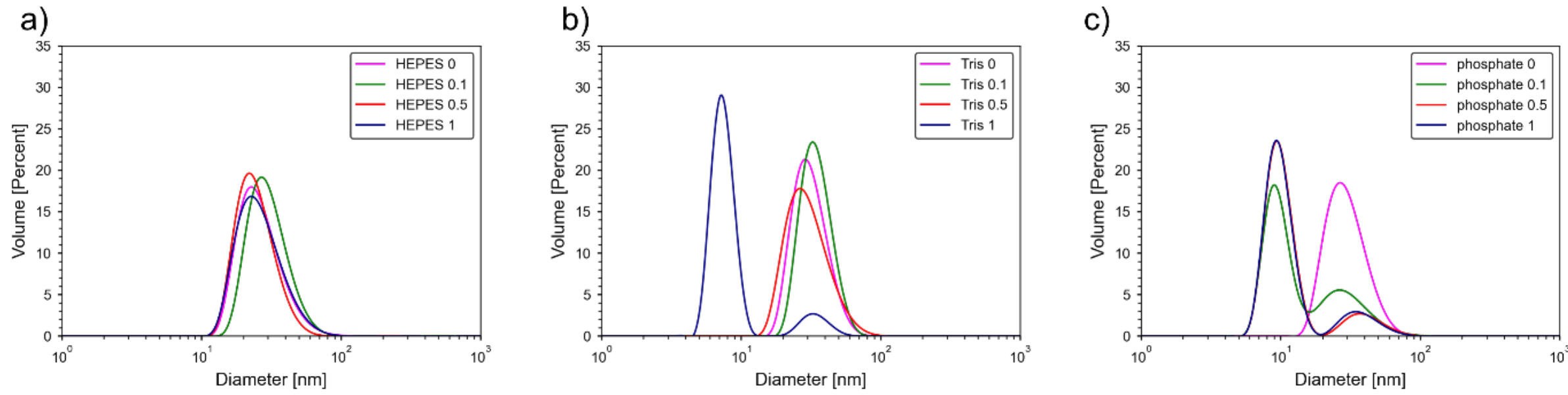

Figure S1. Hydrodynamic diameter distributions by volume of *M. xanthus* encapsulin following reassembly across all buffer and salt conditions. a) HEPES buffer, b) Tris buffer, c) phosphate buffer, each at 0 M NaCl (pink), 0.1 M NaCl (green), 0.5 M NaCl (red), and 1 M NaCl (blue). Under all conditions tested, reassembly produced large assemblies with hydrodynamic diameters consistent with intact encapsulin cages. DLS cannot resolve coexisting $T = 1$ and $T = 3$ populations due to their similar hydrodynamic diameters; SEC was used as the primary method to distinguish between assembly states (Figures 2-5).

Table S1. Hydrodynamic size and dispersity of *M. xanthus* encapsulin. Hydrodynamic size and dispersity of *M. xanthus* encapsulin before disassembly, after disassembly, and following reassembly across all buffer and salt conditions. Z-average hydrodynamic diameters and polydispersity indices (PDI) were determined by dynamic light scattering using cumulant analysis. The native sample (pre-disassembly) shows a monodisperse population consistent with intact encapsulin cages, while disassembly in 8 M urea produces a heterogeneous population of large aggregates. Following reassembly, Z-average diameters in most conditions are consistent with intact cages, though DLS cannot resolve coexisting $T = 1$ and $T = 3$ populations due to their similar hydrodynamic diameters. Buffer abbreviations: Phos, phosphate. NaCl concentrations are given in molar (M).

**Condition**	**Z average diameter (nm) Intensity-weighted**	**Polydispersity Index**
Native	40.5	0.02
Disassembled	487	0.497
HEPES 0 M NaCl	42.2	0.316
HEPES 0.1 M NaCl	42.3	0.354
HEPES 0.5 M NaCl	44.2	0.181
HEPES 1 M NaCl	36.8	0.262
Tris 0 M NaCl	46.5	0.176
Tris 0.1 M NaCl	44.4	0.172

Tris 0.5 M NaCl	43	0.32
Tris 1 M NaCl	344	0.303
phosphate 0 M NaCl	54.2	0.148
phosphate 0.1 M NaCl	43.8	0.305
phosphate 0.5 M NaCl	50.7	0.389
phosphate 1 M NaCl	68.8	0.185

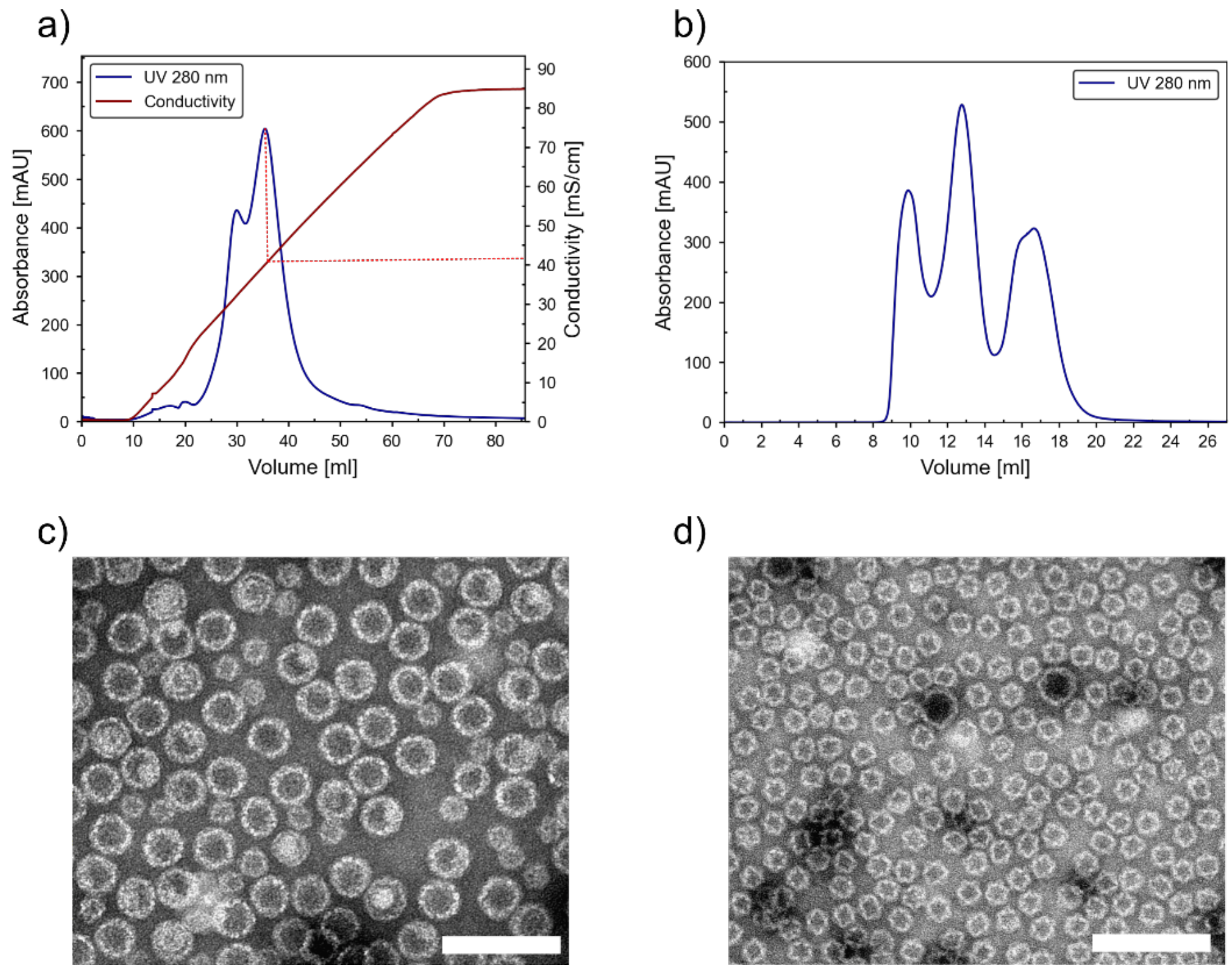

Figure S2. Purification of *M. xanthus* encapsulin and validation of assembly state assignments. a) Anion-exchange chromatography (IEC) elution profile of M. xanthus encapsulin. Absorbance at 280 nm (blue) and conductivity (red) are shown as a function of elution volume. The dashed red lines indicate the collected fraction. b) Size-exclusion chromatography (SEC) profile of the IEC-purified encapsulin on a Superose 6 Increase column, monitored at 280 nm. Three species are resolved: a large particle eluting at approximately 9–10 mL corresponding to $T = 3$ cages, a smaller particle eluting at approximately 13–14 mL corresponding to $T = 1$ cages, and a late-eluting peak corresponding to unassembled subunits or small oligomers. c) Negative-stain TEM image of the $T = 3$ fraction, showing monodisperse intact cages. d) Negative-stain TEM image of the $T = 1$ fraction, showing smaller uniform assemblies. Scale bars, 100 nm. These elution volumes served as reference assignments for SEC analysis of reassembly experiments.

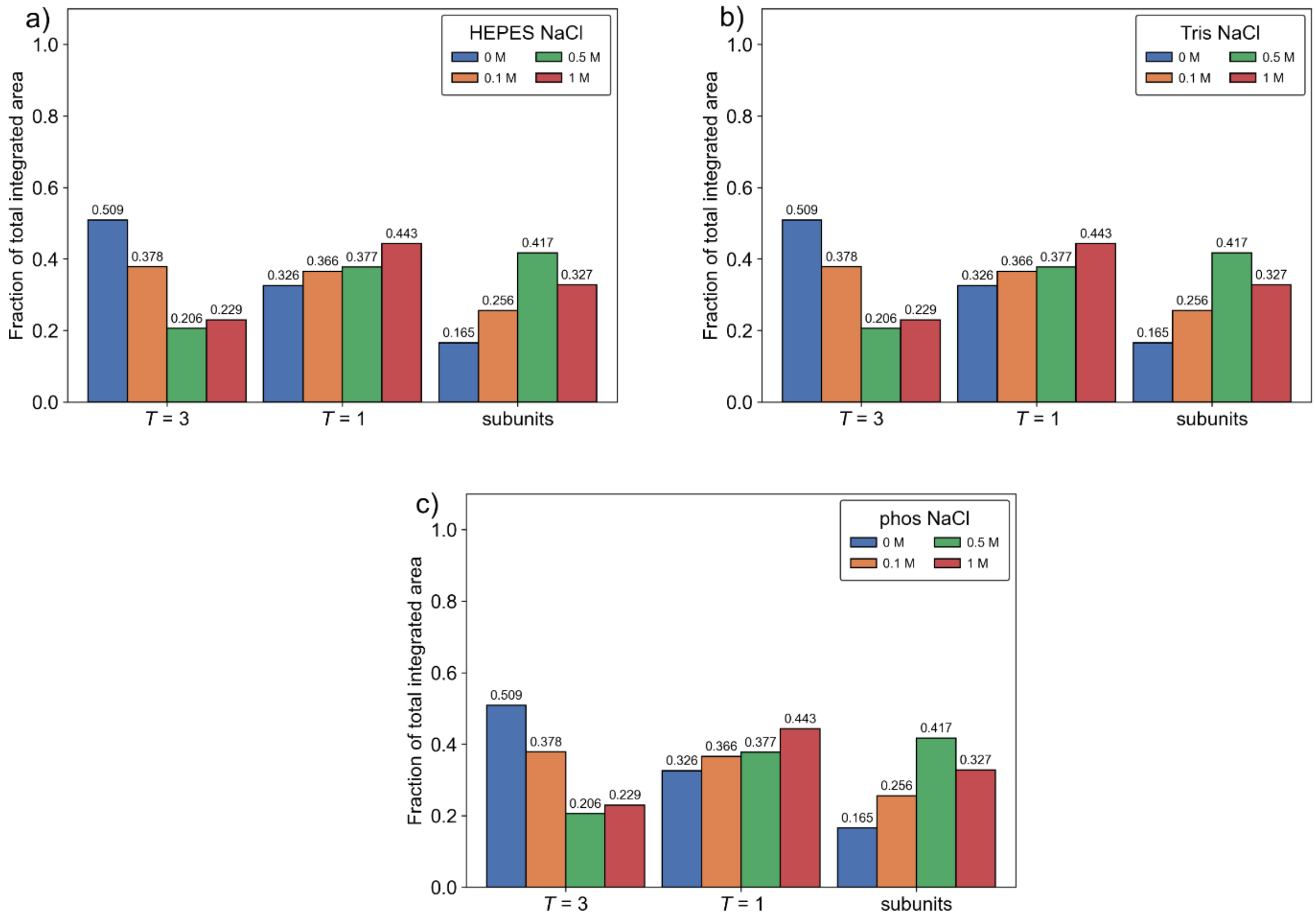

Figure S3. Assembly-state distribution across buffer and salt conditions. Integrated SEC peak areas for the $T = 3$, $T = 1$, and unassembled subunit populations following reassembly in (a) HEPES, (b) Tris, and (c) phosphate buffer at 0, 0.1, 0.5, and 1 M NaCl. Peaks were integrated over fixed elution windows ($T = 3$, 8.5 to 11.5 mL; $T = 1$, 11.5 to 14.5 mL; subunits, 18 to 22 mL) and are expressed as fractions of the total integrated area. In all three buffers the $T = 3$ fraction decreases and the subunit fraction increases with rising NaCl concentration, with Tris retaining the highest $T = 3$ content at each salt concentration. Values at 1 M NaCl carry greater uncertainty because assembly is strongly suppressed and the cage peaks are small.

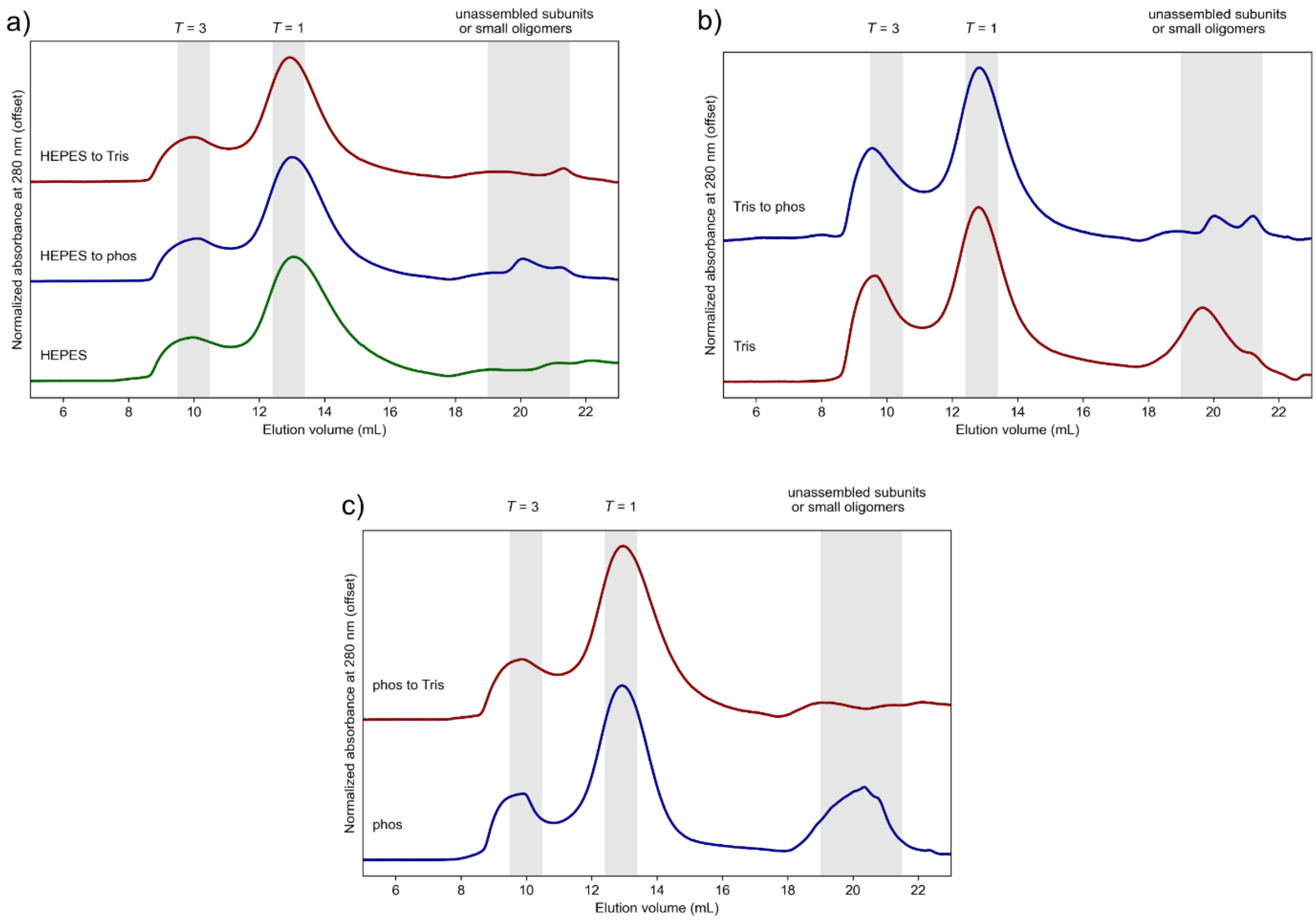

Figure S4. SEC chromatograms before and after post-assembly buffer exchange. Encapsulin was reassembled in (a) HEPES, (b) Tris, or (c) phosphate and then buffer-exchanged into an alternative buffer, with the assembly distribution compared by SEC before and after exchange. a) HEPES-reassembled protein (green) after exchange into phosphate (blue) or Tris (red). b) Tris-reassembled protein (red) after exchange into phosphate (blue). c) Phosphate-reassembled protein (blue) after exchange into Tris (red). Traces are offset vertically for clarity. Shaded regions indicate the elution windows for T = 3 cages, T = 1 cages, and unassembled subunits or small oligomers. In every case the chromatographic profile is retained after buffer exchange, with no redistribution between assembly states. Absorbance was monitored at 280 nm; all buffers were used at 20 mM, pH 7.5.

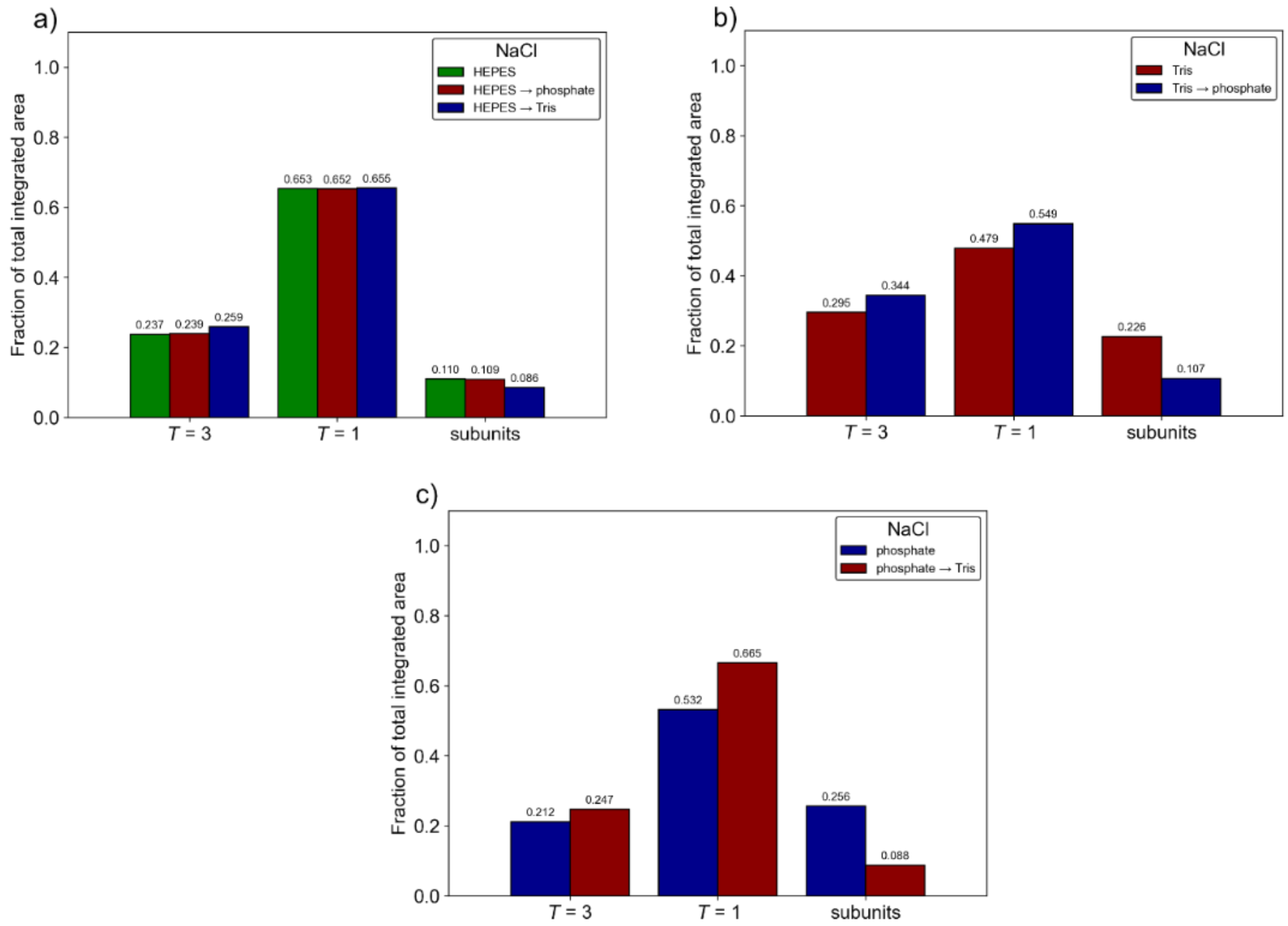

Figure S5. Assembly-state distribution of post-assembly buffer exchange. Encapsulin was reassembled in (a) HEPES, (b) Tris, and (c) phosphate and then buffer-exchanged into an alternative buffer, and the assembly distribution was compared by SEC before and after exchange. Peaks were integrated over the same windows as in Figure S3 and are expressed as fractions of the total integrated area. In both exchange directions, the T = 3 and T = 1 populations increase together while the unassembled subunit fraction decreases, consistent with continued assembly of residual free subunits during handling rather than interconversion between completed cages. Because the samples were split from a single reassembly and filtered separately prior to SEC, absolute peak areas reflect differences in sample handling, and the ratio of T = 3 to T = 1 among assembled cages (Table S2) is the quantity that distinguishes interconversion from continued assembly.

Table S2 (*T* = 3:*T* = 1 ratios). Ratio of T = 3 to T = 1 integrated peak areas before and after post-assembly buffer exchange. Ratios were calculated from SEC peak areas integrated over the windows defined in Figure S3. The ratio remains essentially constant in every condition and does not change in the direction predicted by interconversion between assembled states, confirming that completed cages retain their architecture.

Condition	*T* = 3: *T* = 1 before	*T* = 3: *T* = 1 after
HEPES into phosphate	0.36	0.37
HEPES into Tris	0.36	0.39
Phosphate into Tris	0.40	0.37
Tris into phosphate	0.62	0.63

Table S3. Assembly composition summary. Comparison of assembly properties for the *T* = 3 (PDB: 4PT2) and *T* = 1 (PDB: 7S21) encapsulin shells from *Myxococcus xanthus* as determined by PISA analysis**.** BSA, buried surface area; ΔiG, solvation energy gain on complex formation.

**Property**	***T* = 3 (4PT2)**	***T* = 1 (7S21)**
PDB entry	4PT2	7S21
Assembly	*T* = 3	*T* = 1
Subunits per shell	180	60
Chain IDs	A, B, P (×60)	A-1 to A-60
Number of distinct interfaces	3	180 total
Dominant interface BSA	~1560–1610 Å²	~1123–1127 Å²
H-bonds (dominant interface)	14 (interface 1)	0
Salt bridges (dominant interface)	1 (interface 1)	0

Table S4. Interface summary — *T* = 3 shell (PDB: 4PT2). PISA interface parameters for the three unique interfaces in the *T* = 3 encapsulin shell (PDB: 4PT2). BSA, buried solvent-accessible surface area per chain; ΔiG gain, solvation energy gained upon complex formation; H-bonds and salt bridges are listed per interface. All three interfaces scored CSS = 0.000.

**Interface #**	**Chains**	**BSA Str1 (Å²)**	**BSA Str2 (Å²)**	**H-bonds**	**Salt bridges**	**ΔiG gain Str1 (kcal/mol)**	**ΔiG gain Str2 (kcal/mol)**
1	A–B	1562.9	1612.0	14	1	−8.0	−10.1
2	A–P	645.7	636.2	2	0	−1.6	−3.7
3	B–P	284.8	284.2	0	0	−0.5	−1.0

Interface notes:

**Interface #**	**Notes**
1	Dominant interface; H-bonds and salt bridge (ASP110–LYS270)
2	Secondary interface; 2 H-bonds, no salt bridge
3	Weak tertiary interface; no H-bonds or salt bridges

Note: Interface 1 (A–B) is the dominant inter-subunit contact, burying ~1560–1610 Å² and stabilized by 14 hydrogen bonds and one salt bridge (ASP 110–LYS 270). Interfaces 2 (A–P) and 3 (B–P) are progressively weaker and lack salt bridges, consistent with the hierarchical interface structure discussed in the main text.

Table S5. Interface summary — *T* = 1 shell (PDB: 7S21, representative interfaces). PISA interface parameters for four representative interfaces from the *T* = 1 encapsulin shell (PDB: 7S21, assembly 1; 180 total interfaces reported). Interface #1 is the dominant contact. BSA, buried solvent-accessible surface area per chain; ΔiG gain, solvation energy gained upon complex formation. All interfaces scored CSS = 0.000.

Interface #	Chains	BSA Str1 (Å²)	BSA Str2 (Å²)	H-bonds	Salt bridges	ΔiG gain Str1 (kcal/mol)	ΔiG gain Str2 (kcal/mol)
1	A-27–A-53	1123.5	1126.5	0	0	−9.2	−9.1
31	A-31–A-32	956.9	992.4	0	0	−4.3	−0.5
91	A-25–A-55	734.6	760.2	0	0	−3.4	−4.5
151	A-24–A-3	128.6	129.9	0	0	−0.4	−0.4

Interface notes:

Interface #	Notes
1	Dominant hydrophobic interface; no H-bonds or salt bridges
31	Secondary interface; no H-bonds or salt bridges
91	Tertiary interface; no H-bonds or salt bridges
151	Minimal contact interface; no H-bonds or salt bridges

Note: The dominant *T* = 1 interface (interface #1, representative chain pair A-27–A-53) buries ~1123–1127 Å² per chain with no hydrogen bonds or salt bridges, consistent with primarily hydrophobic stabilization. This contrasts with the *T* = 3 dominant interface, which buries more surface area (~1560–1610 Å²) and is additionally stabilized by 14 hydrogen bonds and a salt bridge. The absence of polar contacts at the *T* = 1 interface supports the interpretation that this assembly is favored under conditions of strong electrostatic screening, where rapid hydrophobic collapse is the primary driver of nucleation.

Table S6. Hydrogen bonds — *T* = 3 Interface 1 (chains A–B). Hydrogen bonds at *T* = 3 interface 1 (chains A–B, PDB: 4PT2). Donor and acceptor atoms are identified by chain, residue number, and atom name.

#	Donor (Structure 1)	Distance (Å)	Acceptor (Structure 2)
1	A:LYS 90 [NZ]	2.44	B:VAL 59 [O]
2	A:LYS 90 [NZ]	2.67	B:SER 60 [O]
3	A:GLN 124 [NE2]	3.04	B:GLY 62 [O]
4	A:ILE 87 [N]	3.81	B:ASP 65 [OD1]
5	A:TYR 89 [OH]	2.36	B:ILE 66 [O]
6	A:TYR 89 [OH]	2.85	B:GLY 68 [O]
7	A:SER 191 [OG]	3.55	B:VAL 166 [O]
8	A:ASN 220 [ND2]	2.10	B:ASN 173 [O]
9	A:SER 112 [OG]	3.78	B:GLU 235 [OE2]
10	A:GLN 124 [OE1]	2.70	B:GLY 62 [N]
11	A:GLN 124 [OE1]	3.66	B:ALA 63 [N]
12	A:TYR 89 [OH]	2.61	B:GLY 68 [N]
13	A:GLN 218 [OE1]	3.77	B:ASN 173 [ND2]
14	A:SER 112 [OG]	2.52	B:LYS 270 [NZ]

Table S7. Salt bridge — *T* = 3 Interface 1 (chains A–B). Salt bridge at *T* = 3 interface 1 (chains A–B, PDB: 4PT2). No salt bridges are present at *T* = 3 interfaces 2 or 3, and none are detected at any *T* = 1 interface.

#	Residue 1 (Structure 1)	Distance (Å)	Residue 2 (Structure 2)
1	A:ASP 110 [OD1]	3.93	B:LYS 270 [NZ]

All interface data were obtained using PDBePISA v1.52. BSA values are per-chain solvent-accessible areas buried at each interface. ΔiG gain values represent solvation energy gained upon complex formation. CSS, Complex Formation Significance Score.
